# Fast Virtual Stenting with Active Contour Models in Intracranical Aneurysm

**DOI:** 10.1038/srep21724

**Published:** 2016-02-15

**Authors:** Jingru Zhong, Yunling Long, Huagang Yan, Qianqian Meng, Jing Zhao, Ying Zhang, Xinjian Yang, Haiyun Li

**Affiliations:** 1 Capital Medical University, Department of Biomedical Engineering, Beijing, 100069, China; 2 Capital Medical University, Beijing Tiantan Hospital, Beijing Neurosurgical Institute, Department of Interventional Neuroradiology, Beijing, 100050, China

## Abstract

Intracranial stents are becoming increasingly a useful option in the treatment of intracranial aneurysms (IAs). Image simulation of the releasing stent configuration together with computational fluid dynamics (CFD) simulation prior to intervention will help surgeons optimize intervention scheme. This paper proposed a fast virtual stenting of IAs based on active contour model (ACM) which was able to virtually release stents within any patient-specific shaped vessel and aneurysm models built on real medical image data. In this method, an initial stent mesh was generated along the centerline of the parent artery without the need for registration between the stent contour and the vessel. Additionally, the diameter of the initial stent volumetric mesh was set to the maximum inscribed sphere diameter of the parent artery to improve the stenting accuracy and save computational cost. At last, a novel criterion for terminating virtual stent expanding that was based on the collision detection of the axis aligned bounding boxes was applied, making the stent expansion free of edge effect. The experiment results of the virtual stenting and the corresponding CFD simulations exhibited the efficacy and accuracy of the ACM based method, which are valuable to intervention scheme selection and therapy plan confirmation.

Intracranial aneurysm, a pathological balloon-like dilatation of cerebral artery wall, is a type of serious cerebrovascular disorder that results from weakening of the artery wall layers, which typically occurs at the Circle of Willis, around or at the bifurcations^1^. Moreover, the rupture of aneurysms will lead to catastrophic complications of high mortality (45–75%) and morbidity, such as subarachnoid hemorrhage^2^. With the rapid development of interventional techniques, the stent implantation for clinical interventional therapy of intracranial aneurysm has become the preferred treatment in many hospitals. However, certain deployed states of stents may increase the risk of aneurysm rupture. Besides, the stent cannot be changed or removed once inserted^3^. Stent implantation is generally performed based on clinical experience without theoretical evaluation for the surgery outcome. How to evaluate the outcomes of stent implantation continuously has been attracting much attention. Image-based computational fluid dynamics (CFD) analysis could potentially provide important insights into the evaluation of treatment outcome but requires fast and realistic representations of stent in deployed states. Therefore, a considerable amount of work has been devoted to developing computational models and methods for the virtual stenting of patient-specific intracranial aneurysms.

During the past decade, many studies on virtual stent deployment mainly focused on *in vitro* experimental simulation and numerical simulation to evaluate stent deployment. Cantón *et al*.^4^ attempted to assess the hemodynamic changes induced by sequential placement of stents using flexible silicone sidewall aneurysm models. However, it is very difficult to simulate the blood flow in patient-specific aneurysms by using *in vitro* models. The Finite Element Method (FEM), on the other hand, can provide an accurate platform for stent deployment simulation. Ding Ma developed a virtual flow diverter (FD) stent deployment method applying finite element analysis^5^,^6^. Bock *et al*.^1^ adopted a finite element method to explore the influence of stent structure and vessel geometry for stent assisted coiling of intracranial aneurysms. However, finite element modeling techniques for virtual stent deployment are time-consuming and not easy to use, making it difficult for clinicians to accurately judge the treatment outcome quickly — FEM has been mainly used to verify the accuracy of other numerical models due to its computational cost. Deformable modeling methods have potential to serve as a fast platform for virtual stent deployment. Florez-Valencia *et al*.^7^ applied a simplex-mesh model with an adapted cylindrical constraint to represent the stent surface for vascular-stent pose simulation. Appanaboyina *et al*.^8^ implemented hemodynamic simulations in patient-specific models with stents that combined body-fitted and embedded grids, and this stent deployment algorithm represents a first attempt at modeling the stent geometry after deployment. Larrabide *et al*.^9^,^10^ proposed a fast virtual stenting (FVS) algorithm based on an extension of simplex deformable models with stent-specific geometrical constraints, which could quickly simulate the expansion of a stent into the patient-specific vascular model of an aneurysmatic region. Egger *et al*.^3^ developed the modeling and visualization techniques for virtual stenting of aneurysms and stenoses, in which the virtual stent was expanded by using an active contour model^11^ in 3D space. Janiga *et al*.^12^ proposed a free-form deformation algorithm for a wall–tight stent deployment^13^,^14^.

In order to develop fast virtual stenting algorithms for patient-specific intracranial aneurysms with timely CFD analysis for surgical planning and treatment decision-making, we proposed a fast image simulation approach that uses an extension of deformable models derived from active contour models^10^ to constrain the virtual stent expanding. Besides, a novel criterion based on axis aligned bounding boxes in self-collision detection^15^ was used for virtual stent deployment termination. Our work achieved the required fitting degree between stent model and patient-specific 3D aneurysm model without edge effect at a minimum computational cost.

## Results

**Figure 1:**
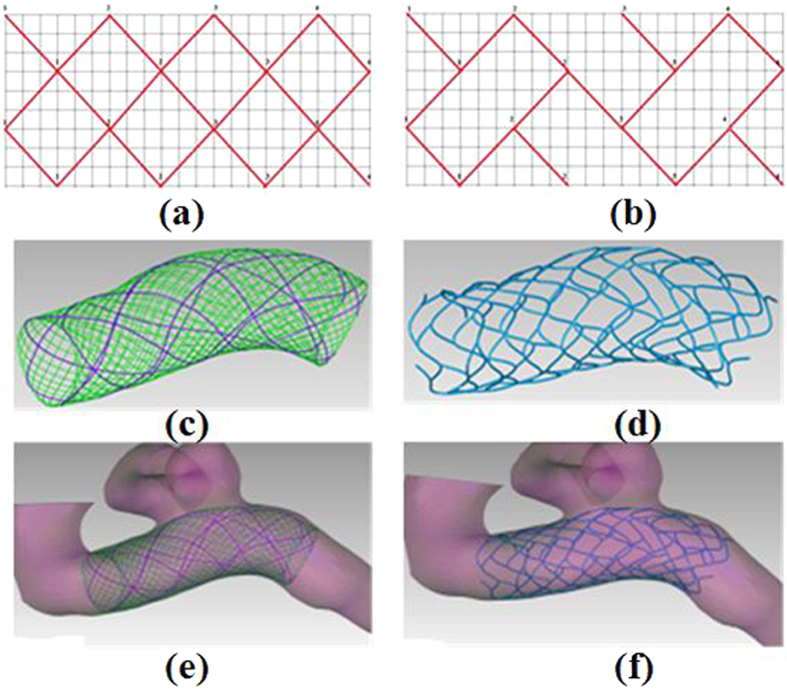
Three-dimensional stent model construction. **(a)** is the mesh of Silk stent model and **(b)** is the mesh of Enterprise stent model. **(c)** is a 3D Silk stent model, where the green lines have a width of 30 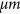 and the purple lines have a width of 50 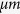. **(d)** is a 3D Enterprise stent model. **(e,f)** are the 3D stent models fitted into the original vessel model with aneurysm.

**Figure 2:**
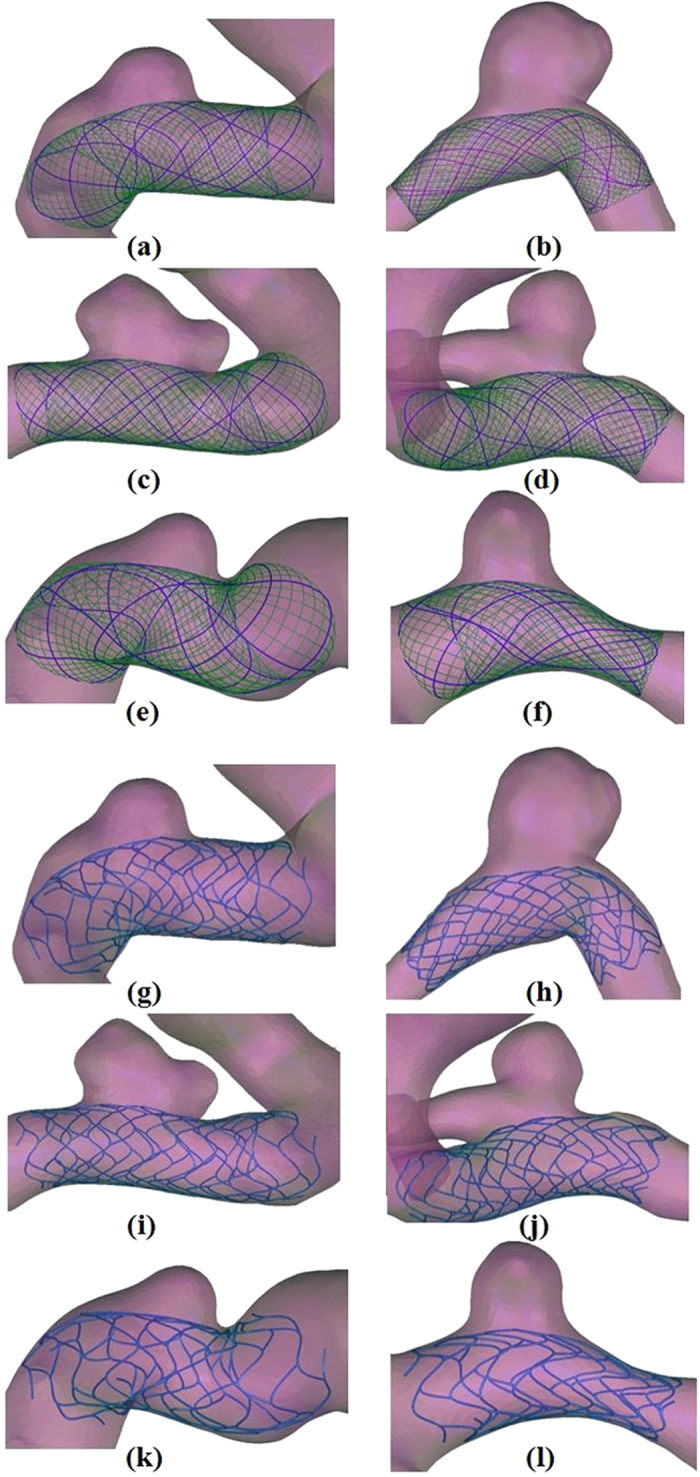
Silk and Enterprise stents deployment in different aneurysms. (**a–f**) Silk stents deployment. (**g–l**) Enterprise stents deployment.

The methodology proposed in this paper has been specifically tailored for its use in the clinical surgical planning process of intervention treatment. Two types of stent designs (Silk stent and Enterprise stent) were adopted to simulate stent deployment in patient-specific aneurysm models derived from real clinical image data. A simplification we made is that stent deployment does not modify the geometry and dimension of the vessel. The three-dimensional stent model fitted into the aneurysm vascular model was constructed according to the stent grid shape (See Fig. 1(a,b)). Two types of stents deployment were simulated on six patient-specific aneurysm models, and the deployment results are shown in Fig. 2.

All simulation computation for stent deployment was executed on a Dell Precision T7500 workstation with two Intel Xeon Processors (2.39 GHz, 2.40 GHz) and 24 GB memory. The computational time depends on the stent node number and vascular triangle number. The average simulation time of the stent deploying within these vessel models was about 3 hours. The average number of iterations was 7. Our proposed method is efficient regarding computational time and easy to implement regarding therapy planning.

**Figure 3:**
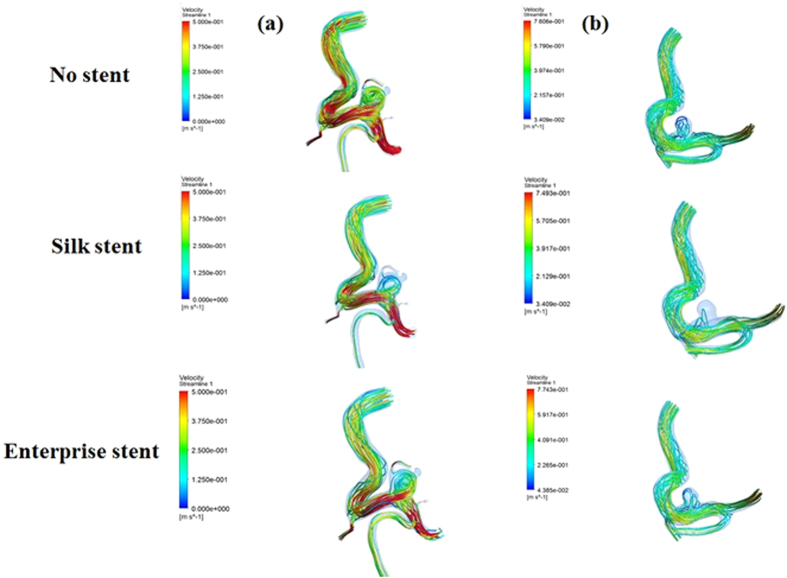
Streamlines depicting the intra-aneurysmal flow patterns before and after the stent deployments. Each column represents a different patient. The first row shows the streamlines without stent implantation. The second row shows the streamlines with the Silk stent implantation. The third row shows the streamlines with the Enterprise stent implantation.

**Figure 4:**
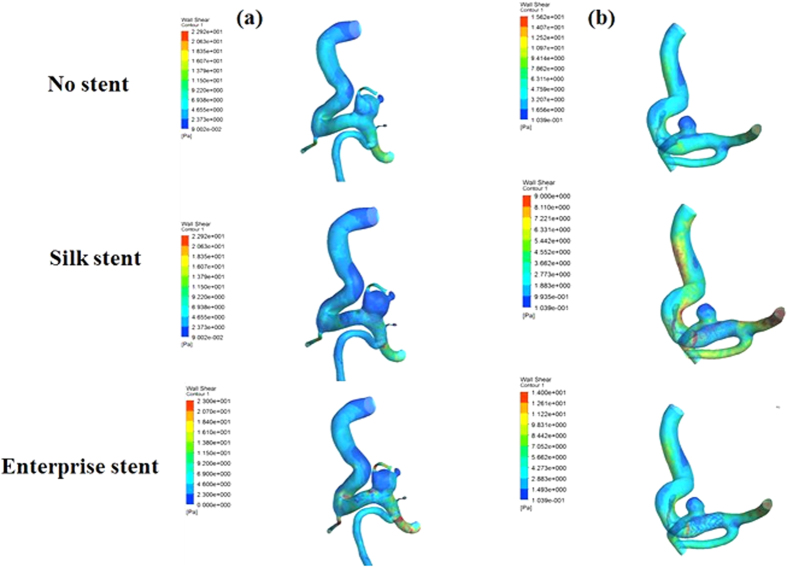
Wall shear stress distributions before and after the stent deployments. Each column represents a different patient. The first row shows the WSS without stent implantation. The second row shows the WSS with the Silk stents implantation. The third row shows the WSS with the Enterprise stents implantation.

Our proposed virtual stenting simulation method allows not only two types of stents to be deployed virtually within patient-specific vascular geometries, but also CFD simulations to be performed before and after the virtual stent deployment, which is necessary when the haemodynamic changes induced by the implantation of the stent need to be investigated. CFD simulation has been successfully used in two types of stenting with patient-specific geometries. As shown in Fig. 3, the blood flow was fairly limited to the aneurysm due to stent implantation, so inflow into aneurysm was reduced and the flow velocity at the aneurysm neck was decreased notably, which were consistent with the results obtained by Appanaboyina *et al*.^8^ and Zhang *et al*.^16^. Besides, the flow pattern became simplex and the vortex in the aneurysmal sac became weaker after the virtual stenting, which was similar to the results by Kim *et al*.^17^ and Zhang *et al*^18^. In addition, Fig. 4 showed a reduction of the WSS (wall shear stress) owing to the presence of the virtual stenting, which can be attributed to flow reduction within the aneurysm^8^,^16^, and a distinct low WSS zone occurred at the aneurysm dome. Furthermore, we found that a Silk stent could induce much more reduction in haemodynamic parameters of aneurysm than an enterprise stent.

## Discussion

The use of stents has expanded the role of endovascular management for wide-necked and non-saccular aneurysms, which are difficult to treat with coil embolization. It is desirable to gain some insights into the surgical procedure and its outcome from a theoretical point of view by simulating the stenting. Virtual stent deployment combined with CFD simulation can provide a lot of useful information for intracranial aneurysm interventional planning. How to reduce the computational cost and keep acceptable accuracy for stent deployment simulation to meet clinical practice requirements remains a challenge.

In this paper, we proposed a fast visualization active contour model based approach to simulating stent deployment for interventional therapy analysis of intracranial aneurysm by using the patient-specific aneurysm model derived from real medical image data. Our proposed algorithms adopted some strategies to ensure the accuracy and efficiency of virtual stenting. Firstly, our virtual stenting algorithms cut off the parent vessel segment and then the aneurysmal sac was shrunk to restore the normal parent vessel by using the aneurysm scaling algorithm proposed in our previous paper^19^, which can avoid the error caused by hand removal of the aneurysmal sac and the mend of the gap. After the above pretreatment, the parent vessel was taken as the target contour of the virtual stent expanding, thus stent mesh nodes could be prevented from moving into the aneurysm sac, and good fitness between the virtual stent contour and the geometrical vessel model could be ensured. Furthermore, the target contour of the stent expanding has been deemed as the main factor affecting the efficiency and accuracy of stent expansion. Existing virtual stenting methods either used the initial aneurysm vessel model^3^, or adopted the pre-processed vessel model, in which the aneurysm sac was removed and the gap was repaired according to experience^20^. In contrast, in our methods, the aneurysmal sac was shrunk to restore the normal parent vessel with our aneurysm scaling algorithm^19^. So the target contour of the stent expanding in our methods has a good match with morphology of the parent vessel.

Besides, we applied a new termination criterion for virtual stent expanding, which was based on axis aligned bounding boxes in self-collision detection^15^. This termination criterion could facilitate the close fitting of the expansion stent mesh to the vessel surface. Secondly, the diameter of the initial stent contour in each slice was set to the maximum inscribed sphere diameter inside the vessel, which reduced the computational cost of image simulation notably and proved to be a feasible solution to the exact positioning of the stent and vascular aneurysm. In particular, the number of nodes in our stent model was almost half the number of other stent models^7^,^8^,^9^,^10^ which further reduced the computation cost at each expanding iteration and then saved total computation time.

FEM based virtual stent deployment algorithms are preferable methods in that they give an accurate description of the stenting procedure, but its set-up and simulation computational time make it impractical for interventional therapy assessment at present. What is more, FEM based approaches are not always applicable to patient-specific geometries, while our proposed ACM based algorithm enables both quick and anatomically accurate stent deployment.

The approaches to virtual stent deployment can be evaluated from the haemodynamic point of view. Our ACM based method for stent deployment has been used to obtain the deployed stent representation, on which the corresponding CFD simulations were carried out, and the changes of our CFD outcomes before and after virtual stent deployment were similar to the related research works^8^,^16^,^17^,^18^. Therefore, to a certain extent, the CFD simulation outcomes indicate efficacy and accuracy of the ACM based method. For the purpose of a further assessment of the ACM based virtual stent deployment method and its limitations, a study comparing it with FEM based method would be appropriate. This is to quantify the deviation that can be attributed to its simplification.

## Materials and Methods

### Materials

Patient’s 3DRA images were obtained from a GE LCV + Digital Subtraction system (LCV; GE Medical Systems) within a 200° rotation at a rate of 8.8 frames/s. The whole 88 projection images were built into a 3D dataset using onboard software on a dedicated GE workstation (Advantage Unix; GE Medical Systems). The raw DICOM files were firstly imported into the Mimics 13.0 application software (Belgium Materialize Company). Then the morphological characteristics of IA were extracted using an image threshold cropping method. The thresholding was adjusted with user interactions. Lastly, the geometry of IA was converted into a triangulated surface model, and the surface contour model was turned into a 3D volume model with application software SolidWorks.2012 (SolidWorks Corp, Concord, MA). All participants gave the informed consents and the Ethics Committee of Beijing Tiantan Hospital Affiliated to Capital Medical University approved the protocol of this study. The procedures were carried out in accordance with the approved guidelines.

## Methods

Our active contour model based virtual stenting algorithms consist mostly of four parts: (1) pre-processing, (2) initial stent contour generation, (3) stent contour expansion and termination, (4) 3D stent model construction.

### Pre-processing

**Figure 5:**
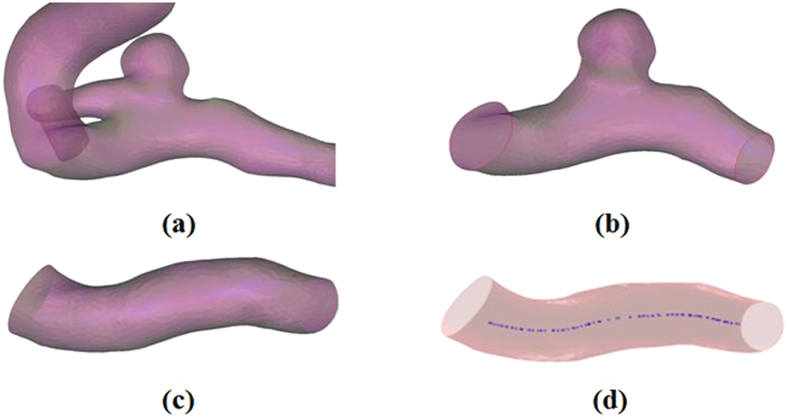
The pre-processing of aneurysm model. **(a)** The original aneurysm vessel. **(b**) The trimmed aneurysm vessel. (**c**) The shrunk aneurysm vessl. (**d**) The extracted vascular center line: the blue line is the center line of blood vessel.

The pre-processing mainly involves cutting off a suitable aneurysm vessel segment from 3D model of the original aneurysm and shrinking the aneurysm to approximate the parent vessel for virtual stent deployment. Firstly, the aneurysm vessel (Fig. 5(a)) was trimmed according to the length of a real stent deployment with application software Geomagic 2012(Raindrop Geomagic, Durham, USA) (Fig. 5(b)). Then the aneurysmal sac was shrunk to approximate the parent vessel by applying the aneurysm scaling algorithm (the details of the scaling algorithm was described in our published paper^19^), as shown in Fig. 5(c).

### Initial stent contour generation

**Figure 6:**
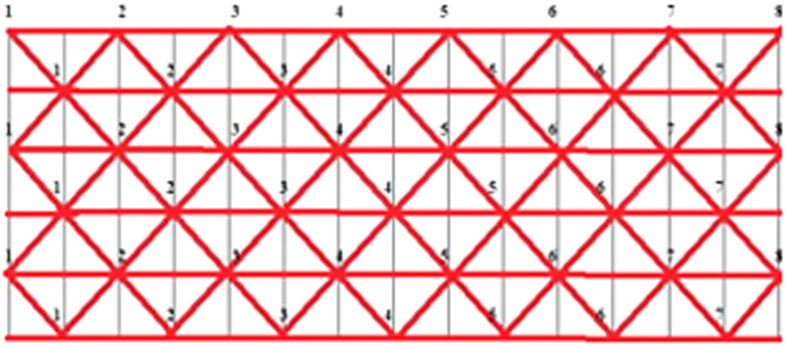
The triangle elements mesh grid.

**Figure 7:**
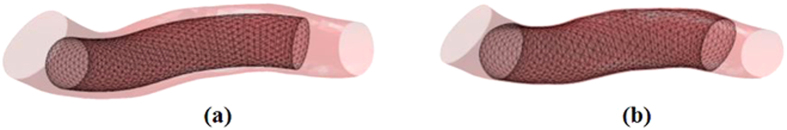
The stent contour before and after the expansion. **(a)** Initial stent contour. **(b)** Final stent contour satisfying the termination conditions.

The initial virtual stent contour produced from a 3D rigid transform was placed along the vessel centerline path (Fig. 5(d)) which was extracted from the parent vessel applying application software VMTK (the vascular modeling toolkit; http://www.vmtk.org). The diameter of the initial stent contour in each slice was set to the maximum inscribed sphere diameter inside the vessel. The stent contour was constructed according to real stent parameters^21^,^22^. The number of nodes in the stent contour depends on the artery’s size and stent design^3^. The coordinates of nodes in the stent contour were computed in Matlab 2010 (The Mathworks, Natick, MA, USA), maintaining relative position among these nodes. All triangular elements (Fig. 6) formed from the nodes made up the initial stent contour (Fig. 7(a)). The initial stent contour and the parent vessel geometry were used in the succeeding steps.

### Stent contour expansion and termination condition

For stenting simulation, the initial stent contour was regarded as deformable contour and the parent vessel (Fig. 5(c)) was served as the object surface. The Active Contour Model (ACM) algorithm^11^,^23^ was applied to drive the deformation of the initial stent contour. The ACM can be expressed by an energy function (equation (1)) consisting of internal and external energies^11^,^24^,^25^,^26^.


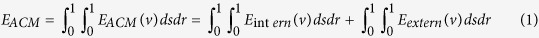


where the 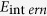 was defined to describe the elastic and smoothing properties of the stent contour and the definition was as follows:


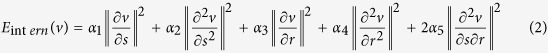


The weighting parameters 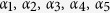 regulate the stiffness properties of the stent contour^22^. And the influence of the 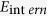 can be simulated by a stiffness matrix which can be solved based on the study by Kass *et al*.^11^ and Cohen *et al*.^26^,^27^.

On the other hand, 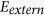 was defined to drive the expansion of the stent contour. Generally, 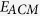 is minimized by building a dynamical system by using a Euler-Lagrange equation which was based on force-based differential equations^11^,^26^. The ACM would be in an equilibrium state when internal forces balance external forces which were corresponding to the internal and external energies, respectively. In our study, the external force 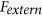 consists of a balloon force 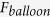 and resistance of the vessel wall force 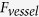.


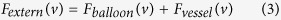


In equation (3), the balloon force was defined as follows^26^:


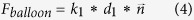


Where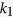 is the weighting parameter of balloon force, 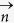 is the unit vector normal to the surface at the stent nodes and 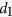 is the shortest distance between the stent nodes and the vessel wall surface.

In addition, the vessel force was defined as follows


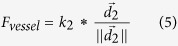


where 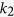 is a weighting parameter of vessel force, and 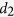 is the vector along the shortest distance with a direction from the current stent node to parent vessel surface.

**Figure 8:**
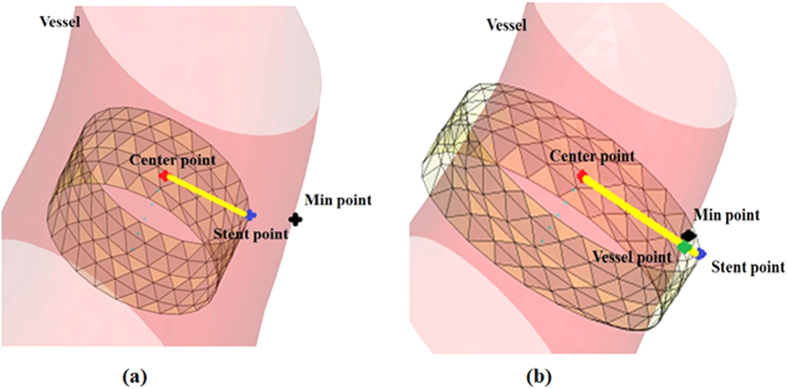
Two cases of stent nodes during the expansion. (**a**) The stent nodes inside the vessel wall. (**b**) The stent nodes outside the vessel wall. The red point is the initial center point; the blue point is a stent node; the black point represents the nearest intersection between the stent nodes and the vascular wall. And the green point indicates the intersection between the ray from the center point to a stent node and the vascular wall.

According the above definitions, the final expansion ACM can be achieved by minimize 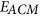, that is to say, the ACM should be in an equilibrium state. Typically, the ACM equilibrium state was gradually approximated via dynamical iterations^26^. Thus in each iteration, the location of a point on the stent contour would be determined based on a modified axis aligned bounding boxes method which was previously used for collision detection^15^,^28^. The detail of the modified method was illustrated as follows (Fig. 8): For any stent node (the blue point in Fig. 8), the center point of the slice, which the current stent node belongs to, is defined as the fiducial point (the red point in Fig. 8). If the connecting line between the center point and the current stent node (the yellow line in Fig. 8) intersects with the vessel surface (the green point in Fig. 8), the stent node is outside the vessel surface, otherwise, it is inside the surface^29^. According to the above criterion and the clinical situation with the consideration of the stent width, the location of the stent point in each iteration would be in one of the following three situations which were corresponding to specific processing principles:

(1) If the location of the current stent node is inside the vessel surface and the shortest distance between the stent node and vessel surface is larger than the threshold 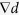, which equal the stent wire width (shown in Fig. 1(c, d)), then the stent node was regarded as a continuation node which would expand in the next iteration.

(2) If the location of the current stent node is inside the vessel wall and the shortest distance between the stent node and vessel wall is equal or smaller than the distance threshold 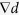, then the stent node was regarded as a termination node. In this situation, the position of this node was relocated in the current iteration according to the following principle:


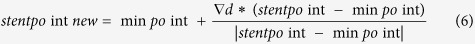


where 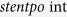 and 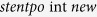 represented the positions of the stent node before and after relocation respectively. 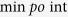 represented the position of the point on the vessel surface which owned the shortest distance to the current stent node.

(3) If the stent node is outside the vessel wall. It is also regarded as a termination node. In this situation, the position of this node was relocated in the current iteration according to the following principle:


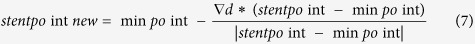


The definitions of all parameters in equation (7) were consistent with those in the equation (6).

In each iteration, the locations of all the stent nodes would be determined. When the percentage of the termination nodes reach 99% compared to the total number of the stent nodes, the iteration ended and stent contour expansion stopped; otherwise the iteration and expansion continued^8^. In the end, the final expanded stent contour could be achieved as shown in Fig. 7(b).

### Three-dimensional stent model construction

Once the expansion stent contour was obtained, the stent wires were connected into 3D wires according to the real stent geometric parameters. In this paper, Silk (Balt International, Montmorency, France) and Enterprise (Cordis Neurovascular, Miami Lakes, FL, USA) stent models were constructed according to the configuration of stent meshes (Fig. 1(a, b)) using application software ABAQUS/Standard 6.8EF (Simulia Corp, Providence, RI, USA) and PTC (Needham, MA). A Silk stent was composed of a dense strut network with 48 wires (8 wires have a diameter of 50 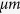 and 40 have a diameter of 30 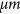)^9^,^21^. The width of 78 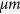 was set for all the wires of the Enterprise stent^9^,^22^. The 3D stent model was implanted into the original patient-specific aneurysm model with application software Geomagic 2012, on which fluid dynamics can be computed^10^. A model was developed for fitting the original vessel model with aneurysm and the 3D stents (Fig. 1(e, f)).

### Computational Fluid Dynamics Simulation

The CFD (computational fluid dynamics) analysis was performed on ANSYS CFX 12.0(ANSYS Inc, Canonsburg, PA, USA) to explore the influence of stent deployment on the hemodynamic parameters of intracranial aneurysms. The aneurysm wall was simplified as a no-slip, no permeable rigid wall^30^. The blood in this study was assumed to be an incompressible Newtonian fluid in laminar flow, with a density of 1060 kg/m3 and a dynamic viscosity of 0.004 Pa. The blood flow varied throughout the cardiac cycle, so the inlet boundary condition was usually assumed to be a pulsating flow. A zero pressure gradient along the flow direction was applied at the outlets^31^. In addition, the CFD computation adopted a parallel computing pattern and ran with two Intel(R) Xeon(R) processors of 2.40 GHz and a RAM of 24.0 GB on a Windows operating system (Version 7).

## Additional Information

**How to cite this article**: Zhong, J. *et al*. Fast Virtual Stenting with Active Contour Models in Intracranical Aneurysm. *Sci. Rep.* **6**, 21724; doi: 10.1038/srep21724 (2016).
